# Deeply divergent sympatric mitochondrial lineages of the earthworm *Lumbricus rubellus* are not reproductively isolated

**DOI:** 10.1186/s12862-015-0488-9

**Published:** 2015-10-05

**Authors:** Iwona Giska, Pierfrancesco Sechi, Wiesław Babik

**Affiliations:** Institute of Environmental Sciences, Jagiellonian University, Gronostajowa 7, 30-387 Kraków, Poland; Institute of Ecosystem Study, Sassari, National Research Council, Traversa La Crucca 3, Regione Baldinca, 07100 Sassari, Italy

**Keywords:** Species delimitation, Cryptic species, RADseq, mtDNA, *Lumbricus rubellus*, Soil diversity

## Abstract

**Background:**

The accurate delimitation of species is essential to numerous areas of biological research. An unbiased assessment of the diversity, including the cryptic diversity, is of particular importance for the below ground fauna, a major component of global biodiversity. On the British Isles, the epigeic earthworm *Lumbricus rubellus*, which is a sentinel species in soil ecotoxicology, consists of two cryptic taxa that are differentiated in both the nuclear and the mitochondrial (mtDNA) genomes. Recently, several deeply divergent mtDNA lineages were detected in mainland Europe, but whether these earthworms also constitute cryptic species remains unclear. This information is important from an evolutionary perspective, but it is also essential for the interpretation and the design of ecotoxicological projects. In this study, we used genome-wide RADseq data to assess the reproductive isolation of the divergent mitochondrial lineages of *L. rubellus* that occur in sympatry in multiple localities in Central Europe.

**Results:**

We identified five divergent (up to 16 % net p-distance) mitochondrial lineages of *L. rubellus* in sympatry. Because the clustering of the RADseq data was according to the population of origin and not the mtDNA lineage, reproductive isolation among the mtDNA lineages was not likely. Although each population contained multiple mtDNA lineages, subdivisions within the populations were not observed for the nuclear genome. The lack of fixed differences and sharing of the overwhelming majority of nuclear polymorphisms between localities, indicated that the populations did not constitute allopatric species. The nucleotide diversity within the populations was high, 0.7–0.8 %.

**Conclusions:**

The deeply divergent mtDNA sympatric lineages of *L. rubellus* in Central Europe were not reproductively isolated groups. The earthworm *L. rubellus*, which is represented by several mtDNA lineages in continental Europe, apparently is a single highly polymorphic species rather than a complex of several cryptic species. This study demonstrated the critical importance of the use of multilocus nuclear data for the unbiased assessment of cryptic diversity and for the delimitation of species in soil invertebrates.

**Electronic supplementary material:**

The online version of this article (doi:10.1186/s12862-015-0488-9) contains supplementary material, which is available to authorized users.

## Background

Species delimitation aims to identify species-level biological diversity while delineating interspecific boundaries and estimating the number of species [1, 2]. The accurate delimitation of species is of paramount importance in numerous fields, including evolutionary biology, systematics, biogeography, conservation biology and many areas of experimental biology [3]. Traditionally, species have been identified based on morphological traits. However, a large portion of the biological diversity may be impossible to detect by relying only on morphological characters [4]. These difficulties are most apparent in taxonomic groups that include closely related, recently diverged species, which form complexes of cryptic species. The morphology-based species delimitation may also severely underestimate the overall diversity in taxonomic groups with morphological uniformity or with a paucity of taxonomically useful morphological characters. Today, many biologists agree that species may be separately evolving metapopulation lineages [5, 6], which is a deliberately loose definition to proceed beyond the unresolvable debate about the species concepts. This lineage-based interpretation of species shifts the focus to genetic data and the other nonmorphological characters. The DNA data may be a source of valuable additional information to develop new and more accurate species delimitation methods that should be used by alpha taxonomists [4]. Distinguishing between the two groups of criteria that are used for species delimitation, the pattern-oriented and the process-oriented criteria, is useful [7]. The pattern-oriented criteria reflect the effect of a lineage existence, e.g., monophyly, diagnosability or formation of distinct genotypic clusters, whereas the process-oriented criteria identify the evolutionary cause of the lineage existence, e.g., the reproductive isolation or occupation of a distinct niche. Species treated as separate evolutionary lineages can be delimited based on these criteria even when the species definition or concept is debated [6]. The use of multiple criteria is recommended to increase the chance to detect recently separated lineages and to obtain clear evidence of the lineages as separate entities [7, 8].

In the past, the molecular identification of species involved primarily mitochondrial DNA (mtDNA) sequences. The most commonly used mitochondrial marker has been Cytochrome Oxidase I (*COI*), which is a standard in the DNA barcoding of animal species, under the sometimes questioned [9] assumptions of low variation within species and high differentiation between species [10]. Within species, high mtDNA differentiation is often observed between allopatric populations; it may or may not be accompanied by a differentiation in the nuclear markers. Sympatric mtDNA divergence is less common, and divergent sympatric lineages often show reproductive isolation and divergence in the nuclear genome (e.g., [11]). However, mtDNA differentiation is often reported to be discordant with the differentiation based on the nuclear genetic markers, and multiple explanations have been proposed for this pattern [2, 12, 13]. Thus, the joint analysis of mitochondrial and nuclear markers in sympatry is more likely to provide a robust test to identify cryptic species and assess species boundaries.

The delimitation of species is of primary importance in the study of belowground fauna. Global biodiversity is determined to a large extent by the belowground communities, and soil is one of the most species-rich terrestrial habitats [14]. A high percentage of species is estimated to remain undescribed for most soil taxa, and this lack of information is likely due to a lack of taxonomic knowledge and expertise, particularly in the case of small body-sized animals [15]. High cryptic diversity has been detected with DNA based methods in soil invertebrates, including springtails [16–18], earthworms [19, 20], mites [21] and centipedes [22]. Some of these soil invertebrates are considered sentinel species in ecotoxicology. The knowledge of their taxonomy, including the cryptic diversity, is critical for the proper design and for the interpretation of ecotoxicological experiments. The divergent evolution of cryptic species may lead to physiological differences, e.g., differential sensitivity to environmental stressors, including pollution. Individuals belonging to separate evolutionary lineages may display significant differences in the sensitivity to toxicants, and such a phenomenon was reported for the evolutionary lineages of the aquatic oligochaete *Tubifex tubifex*, which consisted of five cryptic species*.* The sensitivity to Cd, which was assessed based on the mortality and time to death, differed among these lineages [23]. The distribution of genetic lineages in nature can be expected to be shaped by pollution when the different levels of resistance of the mtDNA lineages to toxicants are considered. Thus, more sensitive lineages would be lost at more polluted sites, as predicted by the genetic erosion hypothesis, which posits the loss of genetic diversity because of pollution [24].

The species of lumbricid earthworms often consist of highly divergent mitochondrial lineages [19, 20, 25, 26]. The epigeic earthworm *Lumbricus rubellus* Hoffmeister, 1843, a sentinel species in ecotoxicology, is found in the UK as two distinct mtDNA lineages, A and B. The mtDNA sequence divergence between these lineages was high, over 8 % at *COI* (K2P distance) and 14 % at *COII* (uncorrected p-distance) [19, 27]. Recently, differentiation in the nuclear markers was also found between individuals from the two mtDNA lineages in sympatry, which implied reproductive isolation and supported the cryptic species hypothesis [27]. Several other deeply divergent mitochondrial lineages have been found within mainland Europe [28]; however, whether these continental lineages are reproductively isolated and represent cryptic species is not known. In Poland, we have identified highly divergent mtDNA lineages in earthworms in sympatry, which represent several of the lineages observed across Europe. Thus, the conditions were favorable to test for reproductive isolation between the mtDNA lineages of *L. rubellus* from continental Europe.

In this work, we tested whether the sympatric mitochondrial lineages of *L. rubellus* that were found in Poland represented cryptic species. We expected the nuclear clustering to be concordant with the sympatric mtDNA lineages if the mtDNA lineages corresponded to reproductively isolated groups. Additionally, this study aimed to estimate the genetic diversity of the *L. rubellus* populations and to compare the haplotypes found in Poland with the mtDNA lineages observed across Europe. The mitochondrial lineages were characterized based on *COI* and *ATP6* sequences, whereas the multilocus genotype data that were generated by the Restriction site Associated DNA Sequencing approach (RADseq) [29] were used to estimate the differentiation in nuclear DNA. The distribution of the divergent mitochondrial lineages was related to environmental data, including data on pollution.

## Results

### mtDNA

**Fig. 1:**
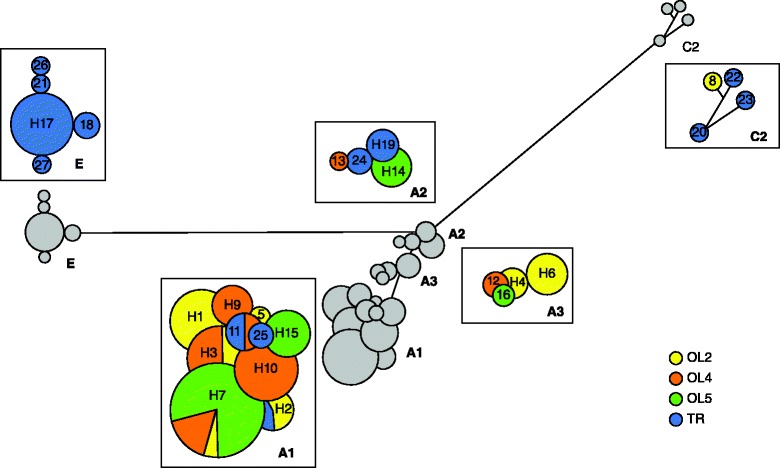
Haplotype network of mtDNA (*COI* + *ATP6*) sequences of *Lumbricus rubellus*. The network shows the divergent mtDNA lineages (A1, A2, A3, C2, and E). Circles represent distinct haplotypes, which are marked with the labels H1-H27 in the enlarged insertions. The size of each circle is proportional to the total number of individuals that showed that haplotype, and the haplotype distributions within the populations are indicated as pie charts. The smallest circle corresponds to *n* = 1

**Fig. 2:**
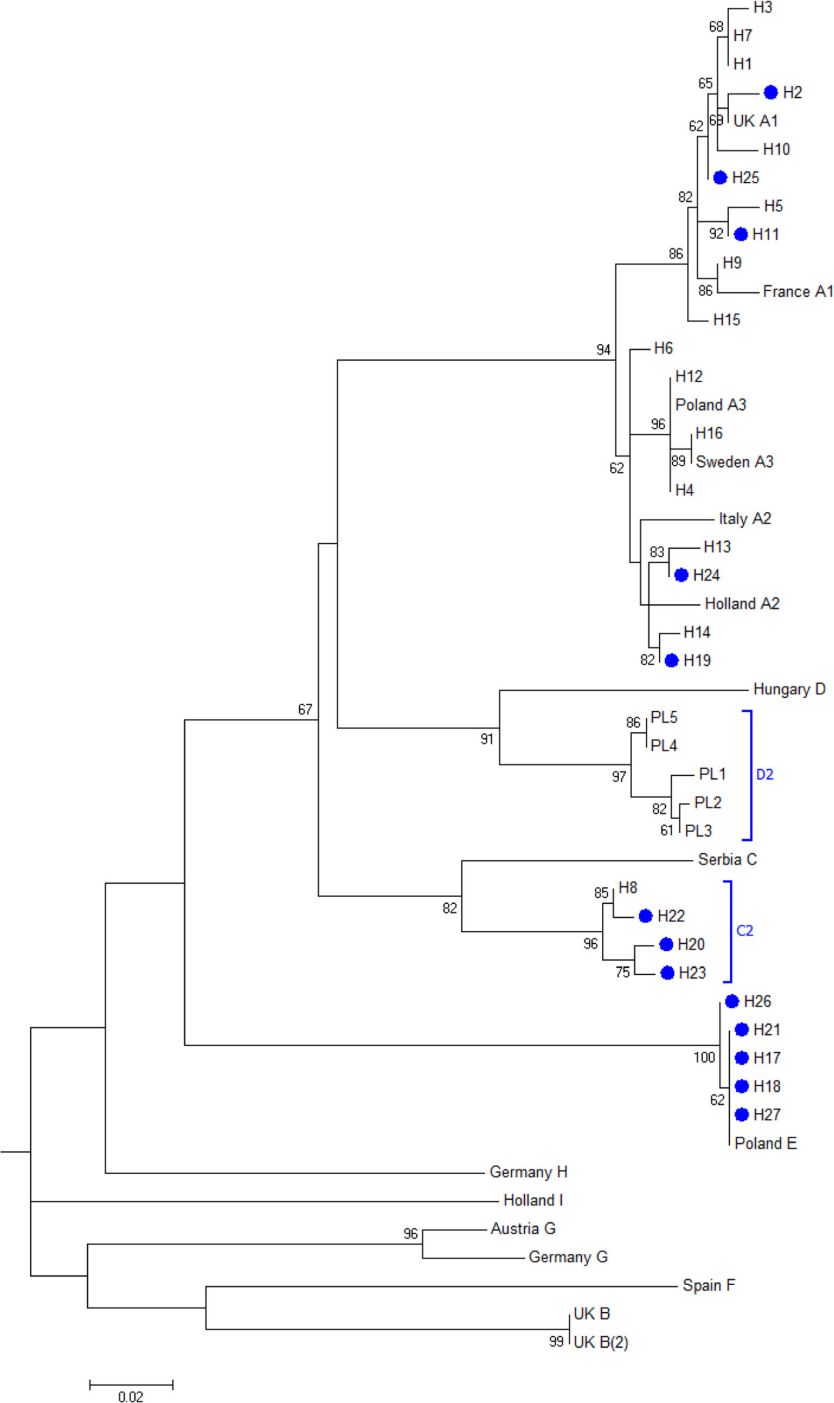
Maximum-likelihood tree based on the *COI* sequences of *Lumbricus rubellus* collected across Europe. Haplotypes observed in the studied Polish populations are marked with the labels H1-H27. Blue circle labels represent the haplotypes found in the TR population. Blue brackets represent the new mtDNA lineages (C2 and D2). The *COI* sequence of *Hirudo medicinalis* [GenBank: EF446709.1] was used as the outgroup to root the tree. Bootstrap percentages ≥ 50 are shown at the branch points

The two analyzed mtDNA fragments totaled 1016 bp (*COI*: 453 bp and *ATP6*: 563 bp). Among the 123 sequenced *L. rubellus* individuals originating from four populations (OL2, OL4, OL5 and TR), 276 polymorphic sites defined 27 unique haplotypes, 10 of which were observed only for one individual (Table 1). *ATP6* showed higher sequence diversity than *COI* (Additional file 1: Tables A1, A2). The haplotypes formed five deeply divergent lineages (Fig. 1); four of the lineages had been previously described (A1, A2, A3, and E), and one lineage was new and named C2 (Fig. 2, Additional file 1: Figure A1). The sequence divergence between the C2 haplotypes and a haplotype from Serbia assigned to the C lineage was 7.5–8.5 %, which led us to distinguish C2 as a separate lineage. An additional mtDNA lineage, which we named D2, was detected in five individuals from another geographic region, and this lineage had not been genotyped with nuclear markers (Fig. 2). The net sequence divergence between the observed lineages was substantial and ranged from 1.3 % between the A2 and A3 lineages to 16 % between the C2 and E lineages (Table 2).

**Table 1:** The mtDNA variation in *Lumbricus rubellus* populations

Site	N	S	H	H_d_	π
OL2	31	174	8	0.815 ± 0.045	0.0277 ± 0.0072
OL4	31	71	7	0.811 ± 0.044	0.0152 ± 0.0029
OL5	31	62	4	0.578 ± 0.081	0.0167 ± 0.0033
TR	30	254	13	0.855 ± 0.056	0.0960 ± 0.0085
*ALL*	*123*	*276*	*27*	*0.923 ± 0.012*	*0.0580 ± 0.0065*

Shown results were based on concatenated mtDNA data (*COI*: 453 bp and *ATP6*:563 bp*).* Site - sampling site, N - number of analyzed individuals, S - number of polymorphic nucleotide positions, H - number of haplotypes, H_d_ - haplotype (gene) diversity (mean ± SD), π - nucleotide diversity (mean ± SD)

**Table 2:** Evolutionary divergence between mitochondrial lineages of *Lumbricus rubellus*

	A1	A2	A3	C2	E
A1		0.032	0.028	0.118	0.157
A2	0.041		0.013	0.114	0.148
A3	0.039	0.023		0.113	0.150
C2	0.133	0.128	0.130		0.160
E	0.162	0.152	0.156	0.170	

Mean pairwise sequence divergence - below diagonal; Net sequence divergence - above diagonal; p-distance

More than one lineage was detected in each studied population, with four lineages co-occurring in the TR population, which also included the most divergent haplotypes (17.5 % uncorrected sequence divergence; Additional file 1: Table A3). The A lineages predominated in the Olkusz area but were found in all studied populations. In contrast to the mtDNA lineages, most mtDNA haplotypes were unique, with only four haplotypes shared by at least two populations and no haplotypes shared by all populations (Fig. 1). Consequently, the mtDNA differentiation (F_ST_) among all population pairs was substantial and highly significant (Table 3). The highest within-population variation, both for the haplotype (H_d_ = 0.855 ± 0.056; mean ± SD) and for the nucleotide (π = 0.096 ± 0.008; mean ± SD) diversity, was found in the TR population. Among the Olkusz populations, the mtDNA diversity increased with the level of pollution, and the OL2 population was the most diverse (Table 1).

**Table 3:** Pairwise genetic differentiation between *Lumbricus rubellus* populations

	OL2	OL4	OL5	TR
OL2	-	0.1518	0.2892	0.1595
OL4	0.1146	-	0.2457	0.1635
OL5	0.1436	0.1828	-	0.2839
TR	0.1278	0.1608	0.1808	-

mtDNA F_ST_ based on haplotype frequency - above diagonal; RADseq F_ST_ based on SNP allele frequency - below diagonal. All values were significant (10,100 permutations; *p* < 0.05, after strict Bonferroni correction)

### RADseq

**Fig. 3:**
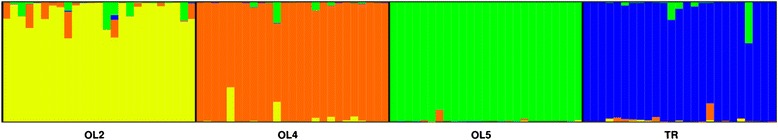
Population genetic structure of *Lumbricus rubellus.* The graph shows the results of the *Structure* analysis of the RAD tags (single SNP selected from each of 1101 RAD tags). Each vertical bar represents a different individual from one of the four populations

**Fig. 4:**
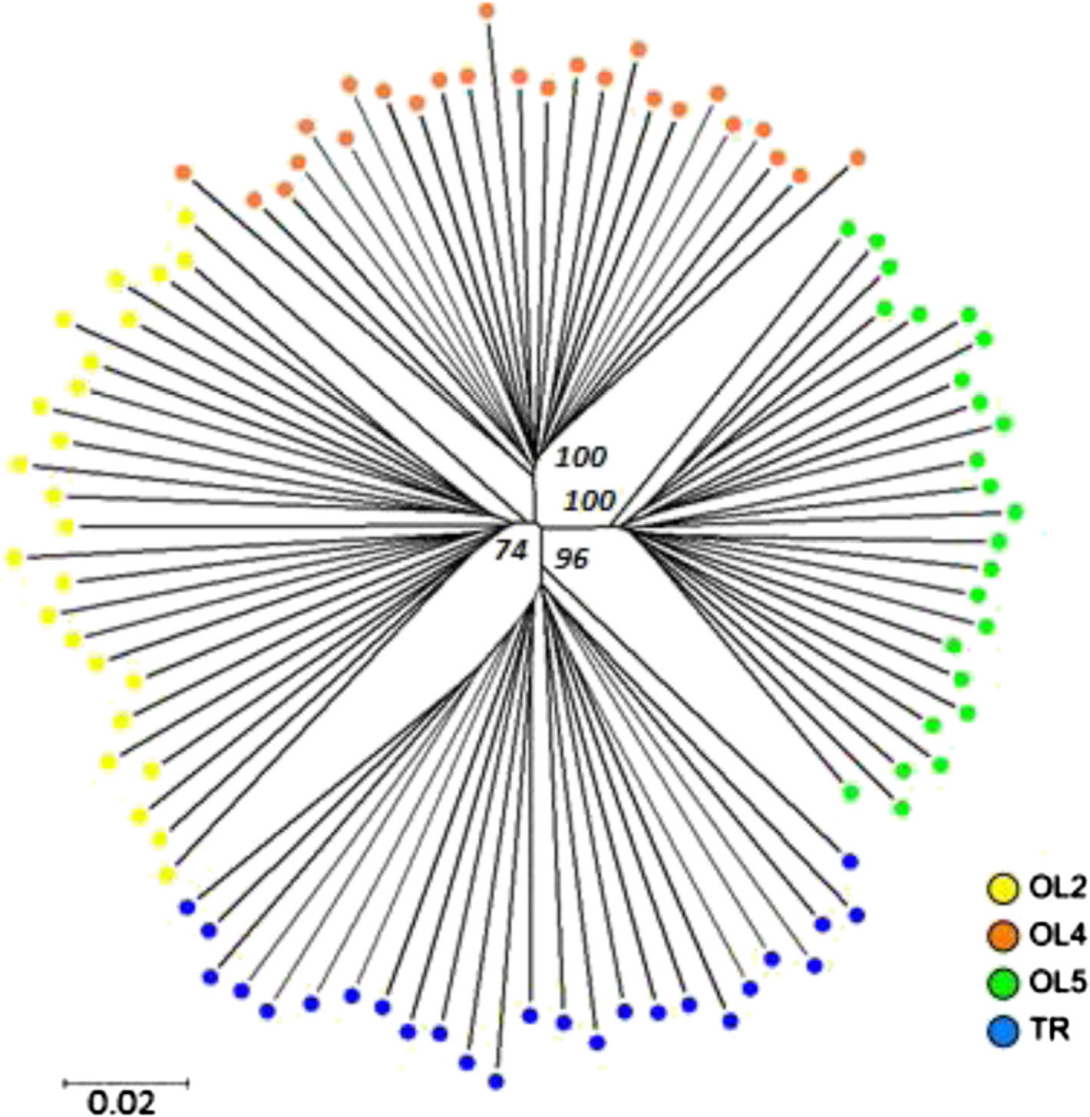
Neighbor-Joining tree generated from the between-individual distance matrix (uncorrected p-distance) based on all 5712 SNPs from the RADseq data. Each dot represents an individual *Lumbricus rubellus* earthworm, and the color represents the population of origin. Bootstrap support values indicate grouping of individuals according to the population of origin

The RADseq data were obtained for 100 individuals, 25 individuals per population. After stringent quality control in *Stacks*, our data set consisted of 1101 RADseq loci (~96,800 bp) that contained 5712 biallelic SNPs. The genetic diversity was highest in the TR population (H = 4.28 ± 0.07; H_d_ = 0.436 ± 0.008; π = 0.0081 ± 0.0002; mean ± SE), which also showed the highest number of private polymorphisms (Sx_TR_ = 1379). Similar to the mtDNA results, the genetic diversity among the Olkusz populations increased with the level of pollution, and the genetic diversity was highest in population OL2 (Table 4). The RADseq-based differentiation between the earthworm populations was significant, and the pairwise F_ST_ ranged from 0.1146 to 0.1828 (Table 3). Although the populations were significantly differentiated in allele frequencies, the overwhelming majority of polymorphic positions were shared among localities, and fixed differences were not observed between the localities (Table 4). The Bayesian clustering identified a clear population structure, the individuals were grouped into four clusters according to their populations of origin with a low level of admixture observed mainly between the neighboring OL2 and OL4 sites (Fig. 3, Additional file 1: Figure A2). The four clusters revealed by *Structure* were recovered also in the Neighbor-Joining tree that was based on the genetic distances between individuals calculated from all 5712 RADseq SNPs (Fig. 4). We did not identify a clear pattern of isolation by distance or a correlation between the level of pollution and the genetic differentiation between the populations (Additional file 1: Figure A3, Additional file 1: Table A4).

**Table 4:** Polymorphism of *Lumbricus rubellus* populations estimated from the RADseq data (1101 RAD tags that contained 5712 SNPs)

Site	H	H_d_	π	S	Sf	Sx	Ss
OL2	3.68 ± 0.06^a^	0.427 ± 0.008^a^	0.0081 ± 0.0002^a^	3239	0	541	2696
OL4	3.23 ± 0.05^b^	0.395 ± 0.008^b^	0.0074 ± 0.0002^ab^	2775	0	372	2403
OL5	2.69 ± 0.04^c^	0.360 ± 0.008^c^	0.0068 ± 0.0002^b^	2284	0	310	1974
TR	4.28 ± 0.07^d^	0.436 ± 0.008^a^	0.0081 ± 0.0002^a^	3816	0	1379	2437

Site – sampling site, H - number of haplotypes (mean ± SE), H_d_ - haplotype (gene) diversity (mean ± SE), π - nucleotide diversity (mean ± SE); S – number of polymorphic sites, Sf – number of fixed differences, Sx – number of polymorphic sites unique for a population, Ss – number of polymorphic sites shared with other populations. Means with different letters are significantly different (t-test; *p* < 0.05, after strict Bonferroni correction)

The clustering of the RADseq data according to the population of origin and not the mtDNA lineage indicated the lack of reproductive isolation between the mtDNA lineages. Although each population contained multiple mtDNA lineages, subdivisions within the populations were not observed in the nuclear genome, not even in the separate *Structure* analysis of the most diverse TR population.

## Discussion

Our analyses of the *L. rubellus* individuals sampled from multiple sites in Poland revealed deeply divergent mtDNA lineages that occurred in sympatry. However, these divergent lineages were not reproductively isolated as evidenced by patterns of clustering in the nuclear data and therefore did not represent cryptic species. The situation we observed in Poland contrasts with that in the UK because the two main mtDNA lineages of *L. rubellus* in the UK, A and B, whose divergence was comparable to that observed in our study, were also differentiated at nuclear microsatellite markers [27]. The morphological data further supported the hypothesis that the two British lineages represent cryptic species [30]. Thus, the level of mtDNA divergence in *L. rubellus* within and between reproductively isolated lineages may be similar. This similarity is not surprising because the divergence at which reproductive isolation evolves varies extensively between and within taxonomic groups [31, 32]. Additionally, Torres-Leguizamon et al. [33] found different patterns of mitochondrial and nuclear structuring in another earthworm species that consist of two highly divergent (8.7 %) mtDNA lineages, *Apporectodea icterica*. This finding suggested the random interbreeding of the mtDNA lineages. Because the nuclear markers of earthworms examined in the present study clustered according to the sample location, we theoretically could have sampled several microallopatric species that share mtDNA lineages. However, the genetic data did not support such a hypothesis. First, fixed differences were not detected among the localities in the nuclear genome; second, the overwhelming majority of polymorphisms were shared among the localities; and third, the signatures of genetic admixture between populations, particularly those separated by small distances in the Olkusz area, were detected. Because our data indicate no reproductive isolation between the lineages, a question arises which processes and mechanisms might explain the presence of highly divergent mtDNA lineages of *L. rubellus* in sympatry? In the following sections, we discuss several plausible, although not mutually exclusive, hypotheses.

The highly divergent mtDNA lineages in Poland might be the result of admixture that followed the postglacial recolonization among previously geographically but not reproductively isolated lineages derived from separate glacial refugia. Such scenario was suggested for the British *L. rubellus* [27] as well as other earthworm species [19, 33]. Recent studies [28, 34] note that cryptic refugium may have occurred on the northwestern coasts of Europe, where one or more of the *L. rubellus* mtDNA lineages could have survived the periods of unfavorable climate during the Pleistocene. The area of present-day Poland may have been colonized from refugia located in central, southern and eastern Europe [35, 36], and such a pattern appears common because zones of contact between divergent evolutionary lineages have been described for multiple taxa in Poland [37–40]. However, the large genetic distances among the mtDNA lineages of *L. rubellus* indicated that their origin predated the Last Glacial Maximum. Nevertheless, multiple cycles of changes in the ranges of the earthworms during the Pleistocene may have caused major changes in the distribution of the ancient mtDNA lineages, which resulted in their sorting into separate refugia. The changes in range may also have prevented the accumulation of reproductive isolation mechanisms between geographically separated populations because of the opportunities for multiple contacts and genetic exchange [41, 42]. The sampling of earthworms in potential refugial areas would provide a direct test of the multiple refugia hypothesis, and a single lineage in a particular area would suggest that the situation observed in Poland resulted from a postglacial admixture. Moreover, admixture might also have contributed to the high nuclear polymorphism detected in our study.

The highly divergent sympatric mtDNA lineages might also be a simple consequence of a large effective population size (*N*e); the estimates of the *N*e may be inflated when coupled with population subdivision and low migration rates [43]. Earthworms are generally considered highly polymorphic organisms that are characterized by a large *N*e and very low migration rates [44]. High intraspecific mtDNA divergence, which often exceeds 5 % in *COI*, is not limited to *L. rubellus* and has been reported in numerous earthworm species, including *Allolobophora chlorotica*, *Aporrectodea rosea*, *Octolasion lacteum*, *Dendrobaena octaedra*, *L. castaneus*, and *L. terrestris* (e.g., [19, 20, 45]). Earthworms appear to also be highly polymorphic in the nuclear genome, although most available estimates are based on microsatellites [25, 27, 46, 47] and are therefore difficult to compare among taxa. However, the genome-wide synonymous nucleotide diversity in *Allolobophora chlorotica* exceeds 1 % [48], which is comparable with the values obtained in our study (0.7–0.8 %). These values might underestimate the true diversity because many polymorphic RADseq loci were filtered out due to the high incidence of missing data. This could result from mutations in the restriction recognition sites but could be also simply due to the random loss of RAD tags during the preparation of RADseq library and random variation in coverage depth. The multiple divergent mtDNA lineages caused by long genealogies in a large population and a high mtDNA mutation rate might be particularly plausible in *L. rubellus*. This species is an obligate cross-fertilizing hermaphrodite: each individual passes its mtDNA to its progeny, which increases the ratio of nuclear to mitochondrial *N*e. For a large *N*e, even colonization from a single refugium could explain our results. Additionally, this hypothesis can be tested by directly sampling populations in multiple putative refugial areas. The comparison of the nuclear diversity between the recently recolonized areas and the refugial areas should indicate whether the colonization was accompanied by a reduction in genetic diversity, as postulated by many models of range expansion [49].

The evaluation of the two hypotheses presented above would require additional sampling and data on genome wide variation and differentiation among populations. However, the RADseq markers are less than ideal for such purposes because a large fraction of the loci are not usable due to the high frequency of missing data. This problem has been previously recognized as a serious issue in highly polymorphic species [50, 51]. Therefore, alternative approaches to estimate nucleotide variation could focus either on the protein-coding genes that harbor extensive synonymous variation and can be analyzed using various targeted resequencing methods [52] or on the ultraconserved [53] or conserved elements [54], which also capture more polymorphic flanking regions.

The high intraspecific mtDNA divergence may be due to an introgression from a related species, as commonly observed in animals [13]. However, the mtDNA sequences of *L. rubellus* found in our study were highly divergent from the sequences of the related species, *L. castaneus* and *L. terrestris*, that were available in GenBank, which eliminated the possibility of introgression from currently known *Lumbricus* lineages. Nevertheless, introgression from an undescribed or extinct lineage remains a viable option.

The multiple divergent mtDNA lineages might also be maintained by natural selection, particularly selection acting in a highly heterogeneous environment like soil. A recent study of Kozancioğlu and Arnqvist [55] suggested that negative frequency-dependent selection (NFDS), a form of balancing selection that favors rare variants, could maintain mtDNA polymorphism [56]. Kozancioğlu and Arnqvist [55] showed an increase in the rare mtDNA haplotype frequency and a decrease in the common haplotype frequency in experimental populations of the seed beetle (*Callosobruchus*) over the course of 10 generations. NFDS is expected under conditions with environmental heterogeneity, genotype-by-environment interactions and competition for resources, which are conditions likely to be common in earthworm populations.

Soil contamination may also affect the distribution of genetic lineages in nature. If the degrees of sensitivity to soil pollution differ among mtDNA lineages, some lineages will be lost in polluted areas, which reduces variation and is consistent with the genetic erosion hypothesis [24]. For example, Andre et al. [57] investigated the highly differentiated populations of *L. rubellus* from a Pb-polluted habitat near Cwmystwyth in Wales, UK. The predominant linage differed by study site depending on the level of contamination, and this pattern supported the loss of distinct mtDNA lineages due to pollution. In our research, four mtDNA lineages occurred in the least polluted TR site. In contrast, the E lineage was not found at any of the polluted Olkusz sites. However, either experimental manipulations or field data from multiple pollution gradients are necessary to demonstrate that individuals carrying the mtDNA of the E lineage are more sensitive to metal pollution. On the other hand, among the three contaminated OL sites, the most polluted site OL2 was characterized by relatively high haplotype and nucleotide diversity and the largest number of the polymorphic sites and private SNPs; therefore, this result did not support the genetic erosion hypothesis and was consistent with a pattern we identified also for the rove beetle *Staphylinus erythropterus*, inhabiting the same gradient [58].

In ecotoxicology, earthworms are used for standard toxicity tests. The recommended and most commonly used species are *Eisenia fetida* and *Eisenia andrei* (e.g., [59, 60]). However, the taxonomy of these species is not clear because of cryptic diversity: The earthworm *E. fetida* has been suggested to be a species complex. Rӧmbke et al. [26] reported two distinct mtDNA *COI* clusters of *E. fetida* that were separated by a p-distance of 11.2 %. Based on the assumption that an uncorrected p-distance > 10 % indicates species level differentiation, these authors hypothesized that *E. fetida* consisted of cryptic species; this result calls the quality and the comparability of ecotoxicological tests into question because cultures of *Eisenia* earthworms are rarely barcoded [26]. Nuclear markers were not applied to confirm the mtDNA clustering of the *E. fetida* reported by Rӧmbke et al. [26], although previous analysis of nuclear *28S* gene indicated possibility that *E. fetida* from Ireland might be a cryptic species [61]. Therefore, the findings of our study are particularly relevant because we showed that high mtDNA divergence, even values exceeding 15 %, did not necessarily indicate the presence of cryptic earthworm species. Thus, in addition to crossbreeding experiments, we recommend the use of multilocus nuclear data to test for cryptic species in *E. fetida*.

The results of our study have consequences for the estimation of the diversity of soil fauna and the delimitation of species. As emphasized by Emerson et al. [62], the soil mesofauna is more diverse than previously thought, and describing the cryptic diversity of soil remains a challenge for ecologists. Thus, the choice of the proper molecular techniques is of crucial importance. The identification of cryptic diversity in the previously mentioned soil invertebrates was often based on a small number of loci [17, 18, 21, 22], although the accuracy of species delimitation is known to depend on the number of loci sampled [63]. Although the cryptic diversity of earthworms has long been a matter of debate, the conclusions about cryptic species remain primarily based on mitochondrial data, which is sometimes complemented with a single nuclear gene or morphological traits (e.g., [45, 61, 64]). In this study, the NGS methods were used to our advantage and showed that genome-wide data supplied valuable knowledge for the study of cryptic diversity.

## Conclusions

The highly divergent mtDNA lineages of the earthworm *Lumbricus rubellus* that sympatrically co-occurred in multiple localities in Poland did not constitute reproductively isolated groups. We concluded that *L. rubellus*, which is represented by several mtDNA lineages in continental Europe, is a single highly polymorphic species rather than a complex of several cryptic species. This study demonstrated the critical importance of multilocus nuclear data for the unbiased assessment of cryptic diversity and species delimitation in soil invertebrates.

## Methods

### Sampling

The earthworms were sampled from the smelting and mining area in southern Poland in the vicinity of the zinc and lead smelter ‘Bolesław’ that is close to Olkusz along a well-studied metal pollution gradient [65, 66]. No specific permissions were required because *L. rubellus* is not a protected species and sampling was not done in a protected area. Sufficient numbers of *L. rubellus* were found at three sites with different levels of pollution: OL2 (50°17′44′′ N, 19°29′27′′ E), OL4 (50°19′05′′ N, 19°30′32′′ E), and OL5 (50°19′46′′ N, 19°32′44′′ E). The soil at these sites was primarily contaminated with Cd, Pb and Zn (Table 5). As a reference, we used the TR site (49°49′14′′ N, 20°01′22′′ E) in Trzemeśnia, which is also in southern Poland and approximately 65 km from the Olkusz area. We sampled only adult earthworms with a fully developed clitellum. The earthworms were collected alive with the use of horse dung traps installed on 15 × 15 m plots. The specimens were washed with distilled water, starved for 48 h and then preserved in 96 % ethanol. Additional earthworms were collected to analyze the metal concentration in the tissues (Table 5). The genomic DNA was extracted from the anterior body segment tissues using the Wizard® Genomic DNA Purification Kit (Promega, Madison, USA).

**Table 5:** Characteristics of the sites at which *Lumbricus rubellus* was sampled

Site	Distance [km]	pH_CaCl2_	OM [%]	Cd [mg kg^−1^]	Pb [mg kg^−1^]	Zn [mg kg^−1^]
OL2	2.5	4.12 ± 0.03	53.5 ± 0.4	49.1 ± 1.1	2 060 ± 37	3 960 ± 54
				*244 ± 120*	*743 ± 234*	*3568 ± 1158*
OL4	5.3	3.46 ± 0.02	54.2 ± 2.0	14.8 ± 0.2	847 ± 38	966 ± 22
				*80.7 ± 33.0*	*209 ± 211*	*1672 ± 1602*
OL5	7.7	4.29 ± 0.01	36.3 ± 0.7	12.1 ± 0.7	708 ± 12	756 ± 11
				*70.2 ± 35.7*	*71.9 ± 75.4*	*1125 ± 653*
TR	~65	5.33 ± 0.04	13.0 ± 0.1	1.77 ± 0.295	65.4 ± 1.10	170 ± 17
				*35.0 ± 17.6*	*1.35 ± 1.50*	*300 ± 72.3*

The distance from the smelter, soil pH, organic matter content at ~10 cm depth (OM %), and metal concentrations [mg kg^−1^ dwt.]: total concentrations in soil (normal font) and concentrations in earthworm tissue (italics), are shown; mean ± SD (soil: *n* = 3; earthworms: *n* = 3–6). Some of the data in the table were obtained from [66]

### Mitochondrial DNA

The fragments of the *COI* and the *ATP6* mitochondrial genes were sequenced. The primers were designed with the Primer3 software [67, 68] based on the conservative fragments of the *L. rubellus* and *L. terrestris* mitochondrial genomes (Additional file 1: Table A5). The PCR reactions contained ~50–150 ng of DNA template, 0.5 μM of each primer, 1X *Taq* buffer with (NH_4_)_2_SO_4_, 1.5 mM of MgCl_2_, 0.2 mM of each dNTP, and 0.75 U of *Taq* polymerase (Thermo Fisher Scientific, Waltham, USA) in a total volume of 15 μl; the reactions were performed under the conditions shown in Additional file 1: Table A5. After the agarose gel visualization and the Exo-AP cleaning (Exonuclease I and Thermosensitive Alkaline Phosphatase; Thermo Fisher Scientific), the PCR products were sequenced using the BigDye® Terminator v3.1 Cycle Sequencing Kit, cleaned with Ethanol/EDTA precipitation and then analyzed on an ABI 3130xl Genetic Analyzer (Applied Biosystems). The raw sequences were aligned with the SeqScape® software (Applied Biosystems).

The *COI* sequences of *L. rubellus* that were sampled from across Europe by members of the Organisms and the Environment research group from the Cardiff School of Biosciences at the University of Cardiff were downloaded from GenBank [GenBank: KP642090-KP612109]. We selected unique sequences that represented the primary haplotype groups, and we used 16 sequences that originated from individuals sampled in 11 European countries (Austria, France, Germany, Holland, Hungary, Italy, Poland, Serbia, Spain, Sweden, and the UK). We also obtained the *COI* sequences from a few Polish individuals for which no RADseq data were generated, and these individuals originated from the OL3 site (located between OL2 and OL4; individuals PL1-PL4; 50°18’29” N, 19°29’45” E) and from central Poland (individual PL5; 51°32′44′′ N, 21°11′22′′ E).

### RADseq

The genomic DNA was extracted from 25 individuals that were randomly selected from each population and was normalized to a concentration of 50 ng/μl using a Qubit® fluorometer. The RAD sequencing libraries were prepared according to the double-digest RADseq method described by Peterson et al. [69]. For each individual, 500 ng of genomic DNA were digested with SphI-HF and MseI restriction enzymes (NEB). After adapter ligation, the individual samples were pooled into four libraries, purified and size selected with the LabChip XT (LabChip XT DNA 300 Assay Kit; PerkinElmer, Waltham, USA). We selected the 346–406 bp fraction to not exceed ~120,000 RAD tags per earthworm. The libraries were amplified in PCR reactions (20 μl) that contained 1X Phusion buffer, 200 μM of each dNTP, 1.0 μM each of PCR1 and PCR2 primers, 0.5 U of Phusion HF polymerase (Thermo Fisher Scientific) and 2.5 μl of the size-selected library under the following conditions: 98 °C for 30 s, followed by10 cycles at 98 °C for 10 s, at 62 °C for 30 s, and at 72 °C for 30 s, and with a final extension at 72 °C for 5 min. The size distribution of the amplified libraries was checked on a Bioanalyzer (HS DNA chips; Agilent Technologies). The libraries were sequenced on an Illumina HiSeq 2000 sequencer (single end, 100 bp) at the Center for Genome Research and Biocomputing of Oregon State University, USA (see Additional file 1 for details).

The raw Illumina reads were analyzed with the *Stacks* software [70, 71]. To avoid incorrect barcode-individual matches, we first removed all reads that had at least one barcode base with quality < 10 Phred. Subsequently, the reads were demultiplexed and cleaned with *process_radtags.pl* (Additional file 1: Table A6), and the SphI recognition site sequence (CATGC) was removed (Additional file 1: Note A1). The loci were reconstructed with *denovo_map.pl* with the following parameters: −*m 4, −M 4, −n 4* (see Additional file 1: Table A7 for the *Stacks* commands and Additional file 1: Table A8 for comparison of results for different Stacks parameters). The *MySQL* database was used for graphical visualization and data filtering. For further analyses, we used the loci found in all four populations that were genotyped in at least 75 % of the individuals of each population, had at least 5× coverage in each individual, and contained no more than 10 SNPs. The sequencing resulted in ~100,000 RAD tags per individual, with mean coverage 28 reads per RAD tag (Additional file 1: Figure A4). Numerous RAD tags were discarded because they were not present in the required % of individuals (Additional file 1: Figure A5).

### Statistical analyses

The earthworms that were sampled from the different sites were assumed to represent local populations. To assess the population genetic diversity in the mtDNA, we estimated the haplotype diversity, nucleotide diversity and the number of polymorphic sites using DnaSP [72]. The measures of population differentiation (mtDNA F_ST_) were calculated based on haplotype frequencies with *Arlequin 3.5* (statistical significance was assessed with 10,100 permutations; [73]). The relationships among the haplotypes were illustrated with a Median-Joining haplotype network that was constructed with *Network 4.6* [74]. To show genetic differences between the identified haplotypes, we calculated the pairwise distances (p-distance and K2P distance) in *MEGA 6* [75]. To relate the haplotypes found in Poland (including haplotypes PL1-PL4 from the additional sites) to the mtDNA lineages observed across Europe, we reconstructed a phylogenetic tree of the Polish and the primary European haplotypes with the Maximum Likelihood (ML) method in *MEGA 6* (HKY + G model; 1000 bootstraps). The model was selected using the Bayesian Information criterion. Additionally, a Bayesian tree was constructed in *MrBayes* (5 mln generations; GTR + G model).

The RADseq data were analyzed with the *populations* program of *Stacks*. The population genetic statistics, such as the number of haplotypes, haplotype diversity and nucleotide diversity, were estimated. The pairwise differentiation between populations (RADseq F_ST_) was estimated with Arlequin based on the SNP allele frequency (statistical significance was assessed with 10,100 permutations of individuals between the populations). The number of polymorphic sites that were shared between the populations (Ss) and unique to the individual populations (Sx) as well as the number of fixed differences between the populations (Sf) were calculated in *mstatspop* [76]. A Bayesian clustering method, implemented in the *Structure* software [77–80], was used to examine the population structure with the estimation of the most likely number of genetically differentiated clusters and the fractions of the individual genotypes that were attributable to each cluster. For the *Structure* analysis, we used one randomly selected SNP per RAD tag (−−*write_single_snp*), which resulted in 1101 loci (Additional file 2: Structure input file). We tested K-values in the range of 1 to 8, with 20 replicates per value. The *Structure* analysis was run with 100,000 burn-in steps and 1,000,000 post-burn-in iterations per run. The admixture model and the correlated allele frequencies model were used. The lambda parameter was set to one (analysis with the estimated value of lambda, λ = 0.4, resulted in the same number of clusters). The optimal K was selected based on the inspection of the change in the probability value of the data for a given K (L(K)), analyzed with Structure Harvester [81], assuming the largest value for a correct K. For an additional examination of the population structuring, all SNPs were used to calculate a pairwise distance matrix between individuals (uncorrected p-distance) to construct a Neighbor-Joining tree in *MEGA 6*.

The isolation by distance was tested with a simple Mantel test. The effect of pollution on the degree of genetic differentiation between the populations was tested with a partial Mantel test while accounting for geographic distance [82]. The tests were performed with the use of the *IBDWS* ([83]; http://ibdws.sdsu.edu/; 10,000 randomizations). The following distance matrices were used in the study: pairwise F_ST_ values, log-transformed geographic distance (straight-line distance), and difference in Cd total soil concentration. To test the differences in the genome-wide genetic diversity between populations, we used t-tests with a strict Bonferroni correction for multiple comparisons.

## Availability of supporting data

The mtDNA sequences of *L. rubellus* generated in this study are available on GenBank under accession numbers: KT731474 – KT731500 (*COI*), and KT731501 – KT731525 (*ATP6*). The RADseq data (*denovo_map.pl* output tsv files) are available in the Dryad Digital Repository at the http://dx.doi.org/10.5061/dryad.8070m. Raw Illumina reads are available at NCBI BioProject number PRJNA296755 (http://www.ncbi.nlm.nih.gov/bioproject/296755) or upon request to the corresponding author.

## Additional files

Additional file 1: **Supplementary materials.** This file includes supplementary materials to the main text: **Note A1.** Detailed description of the Illumina sequencing and *Stack*s analysis of RAD tags. **Table A1.** Variation at *COI* (453 bp) in *Lumbricus rubellus* populations. **Table A2.** Variation at *ATP6* (563 bp) in *Lumbricus rubellus* populations. **Table A3.** Pairwise genetic distances between mtDNA haplotypes H1-H27 found in Poland. **Table A4.** Mantel test and partial Mantel test statistics for *Lumbricus rubellus* populations sampled at sites with different levels of metal pollution. **Table A5.** Information on mtDNA sequence markers of *Lumbricus rubellus* and the PCR conditions. **Table A6.** Quality control of Illumina reads from the HiSeq 2000. **Table A7.** The *Stacks* commands used to process the RADseq data. **Table A8.** Comparison of *Stacks* results for different analysis parameters. **Figure A1.** Bayesian tree based on the *COI* sequences of *Lumbricus rubellus*. **Figure A2.** The Ln P(D) in *Structure* analysis of *Lumbricus rubellus*. **Figure A3.** Relation between the genetic distance (RADseq F_ST_) and the log(geographic distance) in *Lumbricus rubellus* populations; a reduced major axis regression based on the Mantel test. **Figure A4.** Final coverage per RAD tag (mean ± SD) for individual earthworms of *Lumbricus rubellus*. **Figure A5.** Effect of the *Stacks* parameters on the final number of usable RAD tags in *Lumbricus rubellus*. (PDF 650 kb)

Additional file 2: **Structure file.** This is the main *Structure* input file generated by *Stacks* with the use of one SNP per RAD tag. (TSV 438 kb)
