# Supplementary material for: Deeply divergent sympatric mitochondrial lineages of the earthworm Lumbricus rubellus are not reproductively isolated
Source: BMC Evol Biol. 2015 Oct 5;15:217. doi: 10.1186/s12862-015-0488-9 (PMC4595309; doi:10.1186/s12862-015-0488-9)
Supplement: Supplementary file 1 — Supplementary materials. This file includes supplementary materials to the main text: Note A1. Detailed description of the Illumina sequencing and Stacks analysis of RAD tags. Table A1. Variation at COI (453 bp) in Lumbricus rubellus populations. Table A2. Variation at ATP6 (563 bp) in Lumbricus rubellus populations. Table A3. Pairwise genetic distances between mtDNA haplotypes H1-H27 found in Poland. Table A4. Mantel test and partial Mantel test statistics for Lumbricus rubellus populations sampled at sites with different levels of metal pollution. Table A5. Information on mtDNA sequence markers of Lumbricus rubellus and the PCR conditions. Table A6. Quality control of Illumina reads from the HiSeq 2000. Table A7. The Stacks commands used to process the RADseq data. Table A8. Comparison of Stacks results for different analysis parameters. Figure A1. Bayesian tree based on the COI sequences of Lumbricus rubellus. Figure A2. The Ln P(D) in Structure analysis of Lumbricus rubellus. Figure A3. Relation between the genetic distance (RADseq FST) and the log(geographic distance) in Lumbricus rubellus populations; a reduced major axis regression based on the Mantel test. Figure A4. Final coverage per RAD tag (mean ± SD) for individual earthworms of Lumbricus rubellus. Figure A5. Effect of the Stacks parameters on the final number of usable RAD tags in Lumbricus rubellus. (PDF 650 kb) [file 12862_2015_488_MOESM1_ESM.pdf]

Supplementary materials to:

Deeply divergent sympatric mitochondrial lineages of the earthworm *Lumbricus rubellus* are not reproductively isolated

Iwona Giska, Pierfrancesco Sechi, Wiesław Babik

Table A1 Variation at *COI* (453 bp) in *Lumbricus rubellus* populations. Site - sampling site, S - number of polymorphic nucleotide positions, H - number of haplotypes, H_d - haplotype (gene) diversity (mean ± SD), and π - nucleotide diversity (mean ± SD).

**Site**	**S**	**H**	**H_d**	**π**
OL2	67	7	0.791 ± 0.049	0.02514 ± 0.00589
OL4	33	7	0.811 ± 0.044	0.01808 ± 0.00270
OL5	25	4	0.578 ± 0.081	0.01477 ± 0.00268
TR	97	10	0.743 ± 0.081	0.08347 ± 0.00715
ALL	*107*	*22*	*0.880 ± 0.019*	*0.05261 ± 0.00566*

Table A2 Variation at *ATP6* (563 bp) in *Lumbricus rubellus* populations. Site - sampling site, N - number of analyzed individuals, S - number of polymorphic nucleotide positions, H - number of haplotypes, H_d - haplotype (gene) diversity (mean ± SD), and π - nucleotide diversity (mean ± SD).

**Site**	**S**	**H**	**H_d**	**π**
OL2	107	8	0.815 ± 0.045	0.02976 ± 0.00834
OL4	38	7	0.811 ± 0.044	0.01291 ± 0.00307
OL5	37	4	0.578 ± 0.081	0.01821 ± 0.00386
TR	157	12	0.853 ± 0.056	0.10609 ± 0.00956
ALL	*169*	*25*	*0.923 ± 0.011*	*0.06227 ± 0.00726*

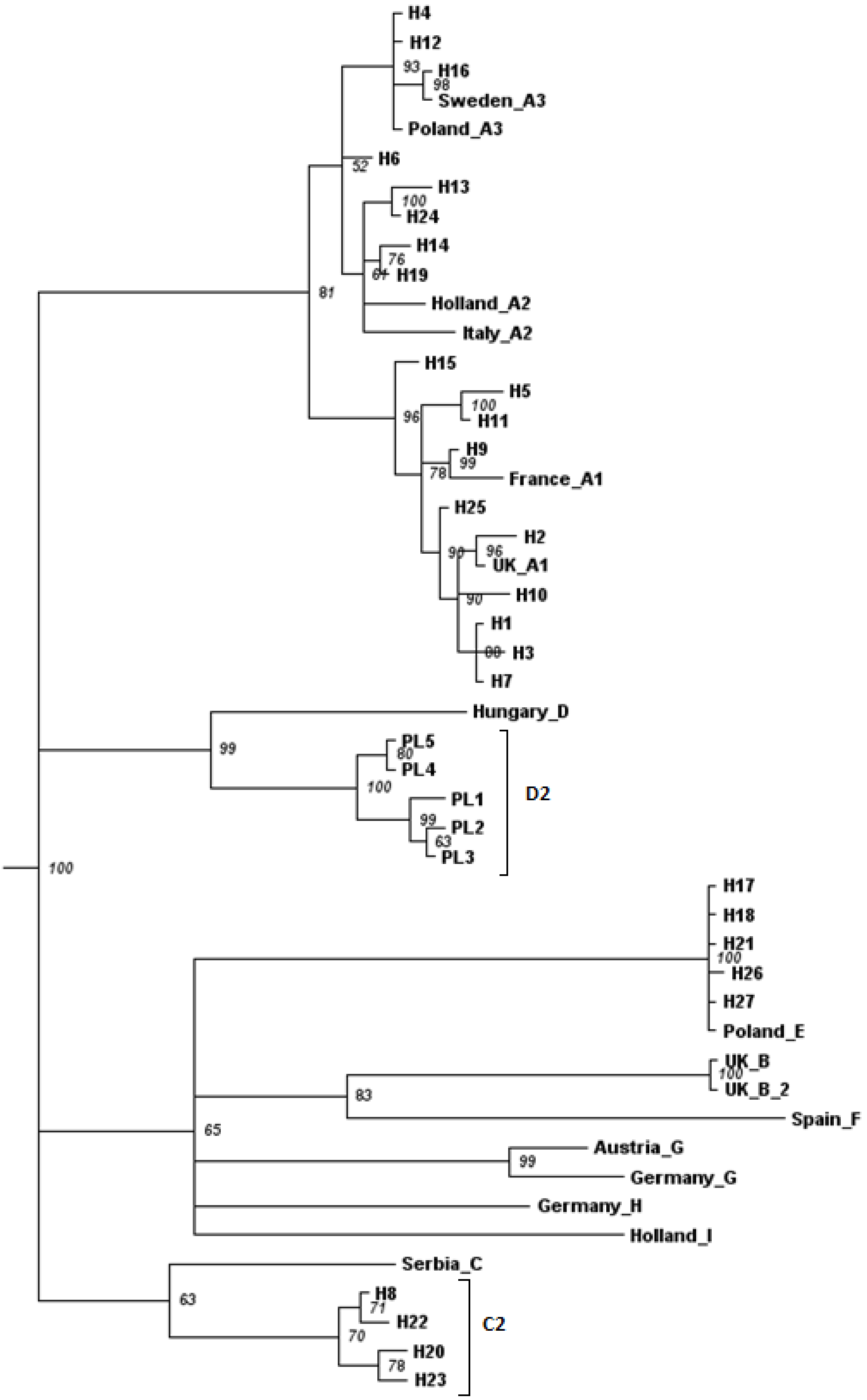

Figure A1 Bayesian tree based on the *COI* sequences of *Lumbricus rubellus*. The posterior probabilities ≥ 50% are shown for each node. Haplotypes observed in the studied populations are marked with the labels H1-H27. The *COI* sequence of *Hirudo medicinalis* (GenBank EF446709.1) was used to root the tree.

Table A3 Pairwise genetic distances between mtDNA haplotypes H1-H27 found in Poland. Uncorrected p-distance below the diagonal (black), and K2P distance above the diagonal (blue); the distances were computed with *MEGA6* based on concatenated *COI* and *ATP6* sequences of *Lumbricus rubellus*.

	**H1**	**H2**	**H3**	**H4**	**H5**	**H6**	**H7**	**H8**	**H9**	**H10**	**H11**	**H12**	**H13**	**H14**	**H15**	**H16**	**H17**	**H18**	**H19**	**H20**	**H21**	**H22**	**H23**	**H24**	**H25**	**H26**	**H27**
**H1**		0.012	0.005	0.040	0.022	0.035	0.000	0.128	0.012	0.012	0.015	0.040	0.046	0.038	0.015	0.046	0.183	0.183	0.032	0.132	0.183	0.135	0.142	0.040	0.005	0.187	0.183
**H2**	0.014		0.017	0.048	0.030	0.043	0.012	0.136	0.020	0.020	0.022	0.048	0.051	0.046	0.022	0.054	0.184	0.184	0.040	0.139	0.184	0.143	0.150	0.048	0.012	0.188	0.184
**H3**	0.007	0.013		0.046	0.027	0.041	0.005	0.135	0.017	0.017	0.019	0.046	0.051	0.043	0.020	0.051	0.190	0.190	0.038	0.138	0.190	0.142	0.149	0.046	0.010	0.195	0.190
**H4**	0.040	0.046	0.041		0.040	0.015	0.040	0.128	0.038	0.043	0.037	0.000	0.027	0.022	0.035	0.005	0.166	0.166	0.017	0.124	0.166	0.135	0.135	0.019	0.035	0.169	0.166
**H5**	0.020	0.024	0.019	0.045		0.043	0.022	0.127	0.019	0.030	0.007	0.040	0.048	0.046	0.022	0.045	0.177	0.177	0.040	0.130	0.177	0.134	0.141	0.043	0.017	0.181	0.177
**H6**	0.036	0.042	0.037	0.022	0.044		0.035	0.129	0.033	0.043	0.035	0.015	0.022	0.017	0.030	0.019	0.163	0.163	0.012	0.125	0.163	0.136	0.136	0.015	0.030	0.167	0.163
**H7**	0.001	0.013	0.006	0.039	0.019	0.035		0.128	0.012	0.012	0.015	0.040	0.046	0.038	0.015	0.046	0.183	0.183	0.032	0.132	0.183	0.135	0.142	0.040	0.005	0.187	0.183
**H8**	0.136	0.132	0.136	0.132	0.137	0.127	0.135		0.125	0.132	0.124	0.128	0.128	0.118	0.115	0.125	0.191	0.191	0.115	0.015	0.191	0.005	0.015	0.125	0.121	0.191	0.191
**H9**	0.009	0.013	0.008	0.037	0.015	0.033	0.008	0.132		0.020	0.012	0.038	0.043	0.035	0.012	0.043	0.171	0.171	0.030	0.128	0.171	0.132	0.138	0.038	0.007	0.175	0.171
**H10**	0.015	0.019	0.014	0.043	0.025	0.040	0.014	0.132	0.014		0.022	0.043	0.054	0.046	0.022	0.048	0.195	0.195	0.041	0.135	0.195	0.139	0.146	0.049	0.012	0.200	0.195
**H11**	0.013	0.017	0.012	0.038	0.009	0.035	0.012	0.133	0.008	0.018		0.037	0.040	0.038	0.015	0.043	0.177	0.177	0.032	0.127	0.177	0.131	0.137	0.035	0.010	0.181	0.177
**H12**	0.041	0.047	0.042	0.001	0.046	0.023	0.040	0.132	0.038	0.044	0.039		0.027	0.022	0.035	0.005	0.166	0.166	0.017	0.124	0.166	0.135	0.135	0.019	0.035	0.169	0.166
**H13**	0.040	0.045	0.041	0.029	0.046	0.019	0.039	0.130	0.037	0.044	0.037	0.030		0.020	0.035	0.032	0.167	0.167	0.015	0.124	0.167	0.135	0.135	0.007	0.040	0.171	0.167
**H14**	0.041	0.047	0.042	0.029	0.049	0.019	0.040	0.127	0.038	0.045	0.040	0.030	0.018		0.027	0.027	0.159	0.159	0.005	0.115	0.159	0.125	0.124	0.012	0.032	0.163	0.159
**H15**	0.015	0.019	0.014	0.041	0.021	0.037	0.014	0.130	0.010	0.020	0.014	0.042	0.039	0.039		0.040	0.159	0.159	0.022	0.118	0.159	0.121	0.128	0.030	0.010	0.163	0.159
**H16**	0.041	0.047	0.042	0.005	0.046	0.023	0.040	0.130	0.038	0.044	0.039	0.006	0.030	0.030	0.042		0.166	0.166	0.022	0.121	0.166	0.132	0.131	0.024	0.040	0.169	0.166
**H17**	0.162	0.164	0.164	0.159	0.164	0.153	0.161	0.166	0.158	0.162	0.161	0.159	0.154	0.153	0.156	0.158		0.000	0.156	0.191	0.000	0.199	0.194	0.163	0.175	0.002	0.000
**H18**	0.161	0.163	0.163	0.158	0.163	0.152	0.160	0.165	0.157	0.161	0.160	0.158	0.153	0.152	0.155	0.157	0.001		0.156	0.191	0.000	0.199	0.194	0.163	0.175	0.002	0.000
**H19**	0.039	0.045	0.040	0.027	0.047	0.017	0.038	0.126	0.036	0.043	0.038	0.028	0.016	0.004	0.037	0.028	0.151	0.150		0.111	0.156	0.121	0.121	0.007	0.027	0.159	0.156
**H20**	0.134	0.130	0.134	0.130	0.135	0.125	0.133	0.022	0.130	0.132	0.131	0.130	0.128	0.123	0.129	0.128	0.170	0.169	0.124		0.191	0.020	0.010	0.121	0.124	0.191	0.191
**H21**	0.163	0.165	0.165	0.160	0.165	0.154	0.162	0.167	0.159	0.163	0.162	0.160	0.155	0.154	0.156	0.159	0.001	0.002	0.152	0.171		0.199	0.194	0.163	0.175	0.002	0.000
**H22**	0.134	0.130	0.134	0.134	0.135	0.131	0.133	0.010	0.130	0.132	0.133	0.134	0.134	0.131	0.128	0.132	0.171	0.170	0.130	0.022	0.172		0.019	0.132	0.128	0.199	0.199
**H23**	0.135	0.131	0.135	0.131	0.134	0.128	0.134	0.023	0.131	0.131	0.134	0.131	0.131	0.128	0.129	0.129	0.173	0.172	0.129	0.021	0.174	0.023		0.131	0.135	0.194	0.194
**H24**	0.038	0.044	0.039	0.026	0.044	0.016	0.037	0.129	0.035	0.042	0.035	0.027	0.003	0.015	0.037	0.027	0.153	0.152	0.013	0.127	0.154	0.133	0.130		0.035	0.167	0.163
**H25**	0.010	0.014	0.009	0.038	0.018	0.034	0.009	0.132	0.007	0.012	0.011	0.039	0.038	0.039	0.013	0.039	0.158	0.157	0.037	0.132	0.159	0.132	0.131	0.036		0.179	0.175
**H26**	0.164	0.166	0.166	0.161	0.166	0.155	0.163	0.168	0.160	0.165	0.163	0.161	0.156	0.155	0.157	0.160	0.003	0.004	0.153	0.172	0.002	0.173	**0.175**	0.155	0.160		0.002
**H27**	0.163	0.165	0.165	0.160	0.165	0.154	0.162	0.167	0.159	0.163	0.162	0.160	0.155	0.154	0.156	0.159	0.001	0.002	0.152	0.171	0.002	0.172	0.174	0.154	0.159	0.004	

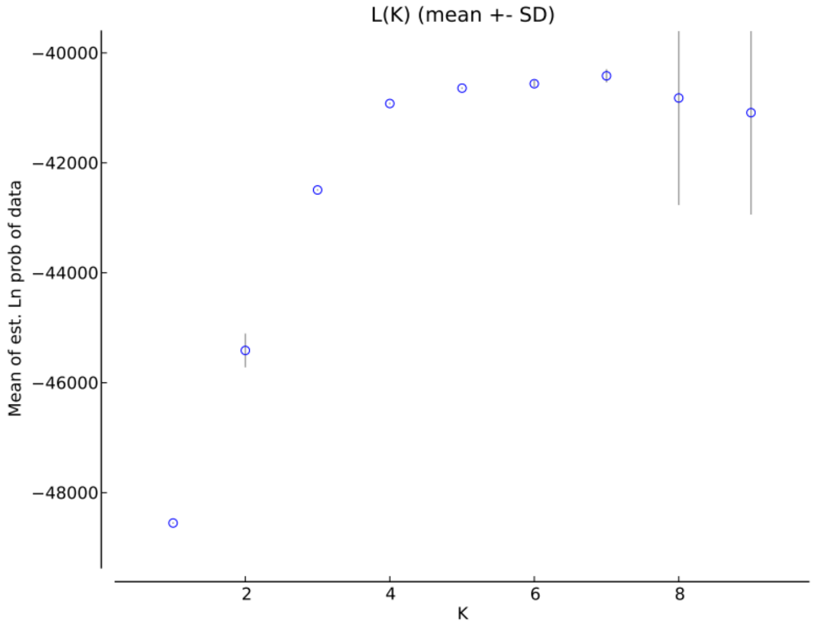

Figure A2 The Ln P(D) in *Structure* analysis of *Lumbricus rubellus*.

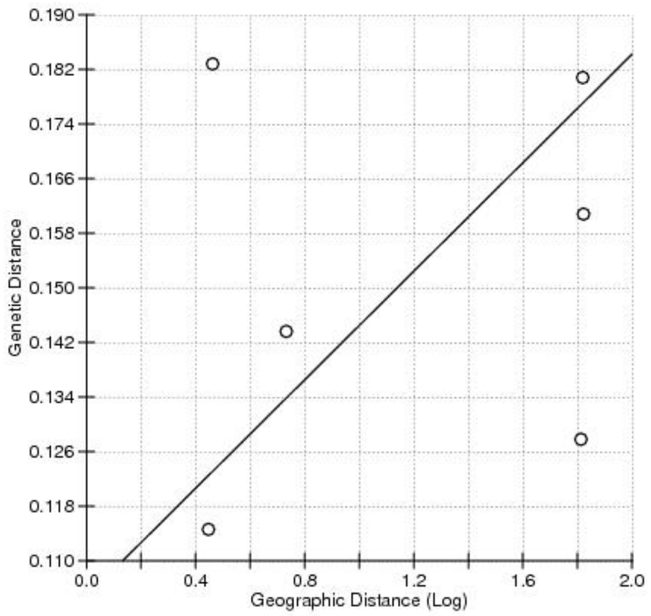

Figure A3 Relation between the genetic distance (RADseq F_{ST}) and the log(geographic distance) in *Lumbricus rubellus* populations; a reduced major axis regression based on the Mantel test.

Table A4 Mantel test and partial Mantel test statistics for *Lumbricus rubellus* populations sampled at sites with different levels of metal pollution.

	**RADseq F_{ST}**
Correlation of genetics and log (geographic distance)	Z = 1.09, r = 0.181, p = 0.363
Correlation of genetics and contamination (indicator) matrix	Z = 19.7, r = -0.887, p = 0.886
Partial corr. of genetics and log (geographic distance), controlling for indicator matrix	r = 0.419, p = 0.324
Partial corr. of genetics and indicator matrix, controlling for geography	r = -0.905, p = 0.839

Table A5 Information on mtDNA sequence markers of *Lumbricus rubellus* and the PCR conditions; the gene, length of the sequenced product after alignment trimming [bp], and PCR primer sequences are shown. The PCR cycle scheme used for sequencing the mitochondrial genes of *Lumbricus rubellus* is presented.

**gene**	**length [bp]**	**primer sequence**	**PCR profile**
COI	453	F1: CCGAATCGAACTAAGrCAAC	95 ^{0}C – 3 min
		F2: GGTCAACAAATCATAAAGATATTGG*	30 cycles:
		R: TCAGAAGAGGTGTTGGTAkAGGA	95 ^{0}C – 30 s
			55 ^{0}C – 30 s
ATP6	563	F: GAGTATCCAAGTCTTGCCATGAT	72 ^{0}C – 1 min
		R: TGkGCGTGrTCrTCTGAGTAT	72 ^{0}C – 10 min

* Folmer universal primer used for some TR individuals for which the F1 primer did not work.

Note A1 Detailed description of the Illumina sequencing and *Stacks* analysis of RAD tags.

A single library run on one HiSeq 2000 lane included 25 individuals that originated from two populations (13 individuals from one population and 12 individuals from another). The populations were distinguished by two indices (6 bp); whereas the individuals were distinguished by 13 different barcodes (5 bp). Because a RAD library is a low-diversity library, the sequencing was performed at a relatively low cluster density (~700 K/mm^2), with a dedicated PhiX lane and a sample PhiX spike in (~15%). In total, the sequencing yielded in 764.4 million (M) reads of *L. rubellus* (Table A6).

The raw Illumina reads were analyzed with the *Stacks* software (Table A7). We discarded all reads that had at least one barcode base with quality < 10 (in the case of one sample, the quality level was increased to 15). This filtering step was performed to ensure the removal of low-quality barcodes because the quality scores were not checked during the *Stacks* analysis. With this filtering, ~23% of the raw reads were discarded. The remaining reads were cleaned and demultiplexed with the *process_radtags.pl* program. Only the reads with the correct barcode and a

high sequence quality were used. We applied the following filters: *-w 0.15*, *-s 10* (default sliding window), *--filter_illumina* (to discard reads failing the Illumina chastity filter), *-c* (to discard reads that contained uncalled bases), *-t 93* (to trim the last three nucleotides), and *-r* (to rescue RAD tags with one sequencing error in the restriction enzyme overhang). After filtering with *process_radtags.pl* we removed the SphI recognition site sequence (CATGC) from all reads, which resulted in a final read length of 88 bp. For each individual, the loci were reconstructed *de novo* with the following parameters of the *denovo_map.pl* program: *-m 4* (required at least four identical reads to form a stack), *-M 4* (allowed a four nucleotide distance between stacks), *-N M+0* (no secondary reads), *-t* (removed highly repetitive RAD tags), *--max_locus_stacks 3* (the maximum number of stacks allowed at a single locus was set to three), *-d* (enabled deleveraging algorithm), and *-n 4* (allowed four mismatches between catalog loci when constructing the catalog). The bounded-error implementation of the maximum-likelihood SNP calling model was used with the upper bound of the error set to a value of 0.05 (results of different α and m values were compared; Table A8). The genotype likelihood ratio test critical value in the SNP calling model was set to 0.1. This implementation resulted in a set of RAD tags with a mean coverage equal to ~28 reads per RAD tag (Figure A4). From this set, we used only the RAD tags that contained no more than 10 SNPs, which were filtered from the *MySQL* database. These parameters were selected after testing various values and accounting for the high polymorphism within *L. rubellus*. For further analyses, we used loci that had at least 5x coverage for an individual, were found in all four populations and genotyped in at least 75% of the individuals of each population (Table A7). We observed a substantial decrease in the usable RAD tags when the *r* parameter was increased (Figure A5).

Table A6 Quality control of Illumina reads from the HiSeq 2000. Raw reads obtained from the Illumina platform, reads retained after filtering sequences with at least one barcode base with a QV < 10 (in the case of samples OL2/II and OL5/II, the quality was increased to 15), reads filtered by *process_radtags.pl* (ambiguous barcodes, failed chastity filter, ambiguous RAD tag, and low QV reads) and reads used for the final analyses (retained reads) are included. Samples marked with the same letter (A, B, C, and D) were pooled and sequenced on one HiSeq lane.

Parameter\Sample	OL2/I [A]	OL2/II [B]	OL4/I [C]	OL4/II [D]	OL5/I [C]	OL5/II [B]	TR/I [A]	TR/II [D]
index	GCCAAT	CTTGTA	CTTGTA	GCCAAT	GCCAAT	GCCAAT	CTTGTA	CTTGTA
raw reads	76,507,384	154,737,876	69,365,166	75,925,083	84,589,867	171,315,380	65,816,468	66,159,114
barcode QV	67,862,301	87,914,574	65,109,910	72,942,177	79,282,359	96,230,896	58,595,180	63,643,136
ambiguous barcodes	26,469,975 (39.0%)	28,530,783 (32.5%)	17,082,714 (26.2%)	17,187,901 (23.6%)	20,770,618 (26.2%)	31,385,971 (32.6%)	22,489,101 (38.4%)	14,658,013 (23.0%)
failed chastity filter	3,998,262	8,318,041	3,928,411	4,024,905	4,967,865	9,340,745	3,430,429	3,467,755
ambiguous RAD tag	209,655	154,546	214,674	190,451	212,073	216,087	148,705	202,056
low QV reads	2,081,921	1,990,208	2,436,512	2,737,386	2,970,886	2,229,686	1,796,958	2,363,596
retained reads	35,102,488 (45.9%)	48,920,996 (31.6%)	41,447,599 (59.8%)	48,801,534 (64.3%)	50,360,917 (59.5%)	53,058,407 (31.0%)	30,729,987 (46.7%)	42,951,716 (64.9%)

Table A7 The *Stacks* commands used to process the RADseq data.

program	command
process_radtags.pl	`process_radtags -p … -b … -o … -c -q -r -t 93 -e sphI \`<br>`-i fastq --barcode_dist 1 --filter_illumina`
denovo_map.pl	`denovo_map.pl -T … -m 4 -M 4 -n 4 -N M+0 -t -H -B … -b … \`<br>`-X "ustacks:-d" -X "ustacks:--max_locus_stacks 3" \`<br>`-X "ustacks:--model_type bounded" \`<br>`-X "ustacks:--bound_high 0.05" -X "ustacks:--alpha 0.1"`
export_sql.pl	`export_sql.pl -D … -b … -f … -o tsv -F snps_u=10`
populations	`populations -b … -P … -M … -r 0.75 -p 4 -m 5 -W …`

Table A8 Comparison of *Stacks* results for different analysis parameters. alpha = the genotype likelihood ratio test critical P value of the SNP calling model (in *denovo_map*.pl), m = minimum stack depth required for individuals at a locus (in *populations*), S = polymorphic sites, π = nucleotide polymorphism (SE).

alpha	m	# loci	S				π			
			OL2	OL4	OL5	TR	OL2	OL4	OL5	TR
0.1	5	1101	3239	2775	2284	3816	0.0081 (0.0002)	0.0074 (0.0002)	0.0068 (0.0002)	0.0081 (0.0002)
	10	723	2110	1803	1493	2524	0.0082 (0.0002)	0.0074 (0.0002)	0.0070 (0.0002)	0.0082 (0.0002)
0.05	5	1103	3238	2774	2287	3815	0.0081 (0.0002)	0.0074 (0.0002)	0.0068 (0.0002)	0.0081 (0.0002)
	10	723	2106	1801	1494	2521	0.0082 (0.0002)	0.0074 (0.0002)	0.0070 (0.0002)	0.0082 (0.0002)
0.01	5	1103	3229	2762	2279	3802	0.0081 (0.0002)	0.0074 (0.0002)	0.0068 (0.0002)	0.0081 (0.0002)
	10	724	2102	1796	1492	2518	0.0081 (0.0002)	0.0074 (0.0002)	0.0070 (0.0002)	0.0081 (0.0002)

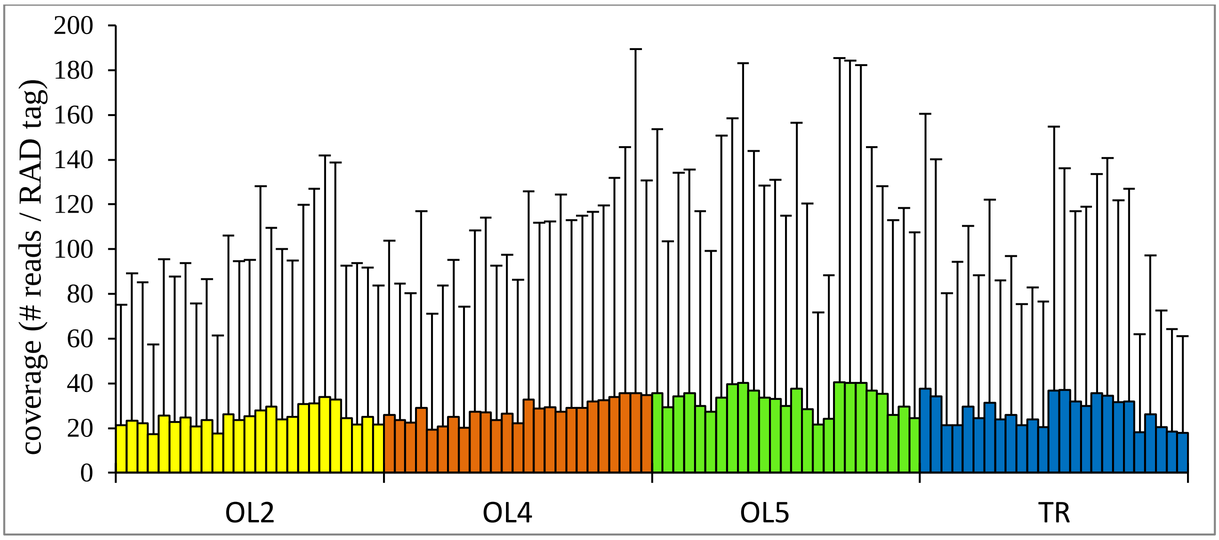

Figure A4 Final coverage per RAD tag (mean ± SD) for individual earthworms of *Lumbricus rubellus*. The coverage was calculated after merging stacks in *denovo_map.pl*. The individuals are represented by single bars, and the colors represent the populations.

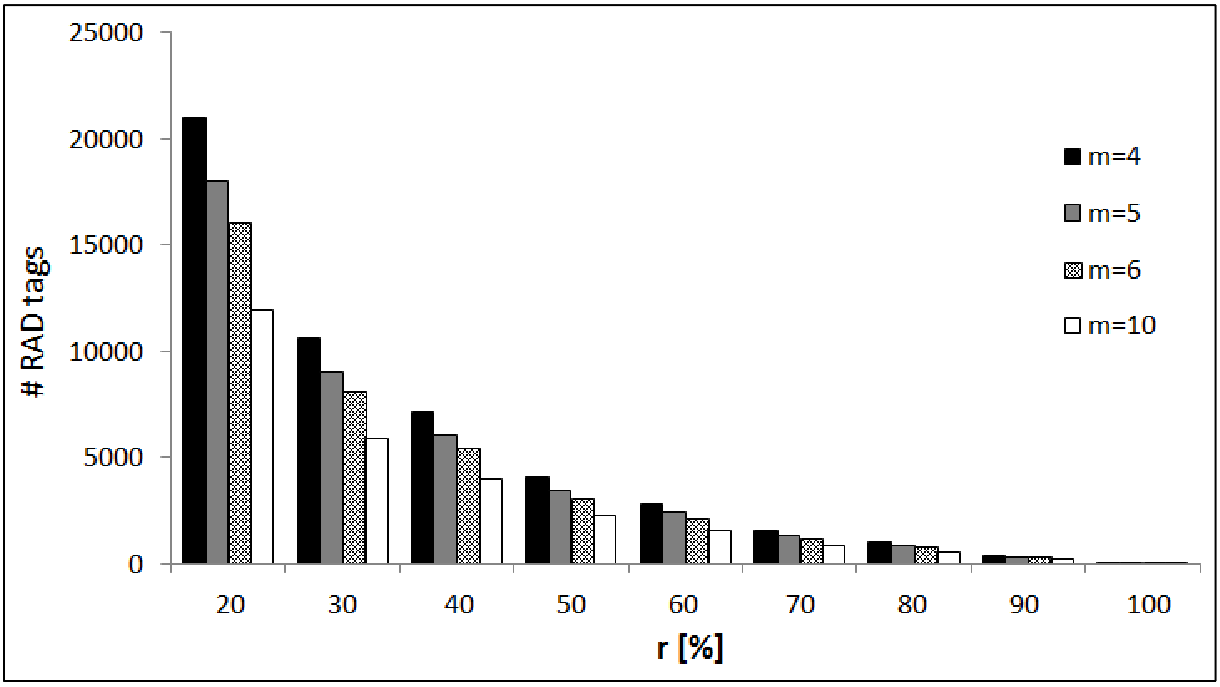

Figure A5 Effect of the *Stacks* parameters on the final number of usable RAD tags in *Lumbricus rubellus*. r = minimum percentage of individuals in a population required to process a locus for that population, and m = minimum stack depth required for individuals at a locus.
